# Circadian disruption-induced microRNAome deregulation in rat mammary gland tissues

**DOI:** 10.18632/oncoscience.157

**Published:** 2015-04-26

**Authors:** David Z. Kochan, Yaroslav Ilnytskyy, Andrey Golubov, Scott H. Deibel, Robert J. McDonald, Olga Kovalchuk

**Affiliations:** ^1^ Department of Biological Sciences, University of Lethbridge, Lethbridge, AB, Canada; ^2^ Canadian Centre for Behavioural Neuroscience, Department of Neuroscience, University of Lethbridge, Lethbridge, AB, Canada

**Keywords:** breast cancer, circadian disruption (CD), microRNA (miRNA), epigenetics

## Abstract

Breast cancer is the most common malignancy affecting women worldwide, and evidence is mounting that circadian-disruption-induced breast cancer is a warranted concern. Although studies on the role of epigenetics have provided valuable insights, and although epigenetics has been increasingly recognized in the etiology of breast cancer, relatively few studies have investigated the epigenetic link between circadian disruption (CD) and breast cancer. Using a proven photoperiod-shifting paradigm, differing degrees of CD, various tissue-extraction time points, and Illumina sequencing, we investigated the effect of CD on miRNA expression in the mammary tissues of a rodent model system. To our knowledge, our results are the first to illustrate CD-induced changes in miRNA expressions in mammary tissues. Furthermore, it is likely that these miRNA expression changes exhibit varying time frames of plasticity linked to both the degree of CD and length of reentrainment, and that the expression changes are influenced by the light and dark phases of the 24-hour circadian cycle. Of the differentially expressed miRNAs identified in the present study, all but one have been linked to breast cancer, and many have predicted circadian-relevant targets that play a role in breast cancer development. Based on the analysis of protein levels in the same tissues, we also propose that the initiation and development of CD-induced breast cancer may be linked to an interconnected web of increased NF-κB activity and increased levels of Tudor-SN, STAT3, and BCL6, with aberrant CD-induced downregulation of miR-127 and miR-146b potentially contributing to this dynamic. This study provides direct evidence that CD induces changes in miRNA levels in mammary tissues with potentially malignant consequences, thus indicating that the role of miRNAs in CD-induced breast cancer should not be dismissed.

## INTRODUCTION

In 2007, the International Agency for Research on Cancer concluded “shiftwork that involves circadian disruption is probably carcinogenic to humans” [1]. Studies conducted before and since this claim have all contributed evidence that breast cancer induced by circadian disruption (CD) is a warranted concern. Amongst the supporting evidence are aberrant, nocturnal melatonin levels contributing to breast cancer development, a role of circadian-relevant genes in breast cancer, and case study findings showing indirect evidence that night-shift workers are at higher risk of developing breast cancer [1-5]. Although many studies have been conducted on the potential mechanisms in CD-induced breast cancer, very few of these studies have investigated the epigenetic links that may be involved. One of these potential epigenetic links is the activity of miRNAs, and the role of these small RNAs in both circadian rhythms and the etiology of breast cancer have been increasingly recognized [6-8].

Mature miRNAs are abundant, small, single-stranded noncoding RNAs that are potent regulators of gene expression [9]. Approximately 22 nts long, mature miRNAs associate with the RNA induced silencing complex (RISC) and target the 3` UTR region of target mRNAs, resulting in gene degradation or suppression, depending on the level of complementarity between the miRNA and its target [7]. In mammals, through this mechanism a single miRNA can target multiple genes and influence a broad range of cellular processes related to cancer [7]. Acting as tumour suppressors, tumour promoters referred to as oncomiRs, or both depending on the degree of malignancy, miRNAs can influence the progression of cancer through various mechanisms such as cellular differentiation, proliferation, and apoptosis [6, 7].

In addition to playing a role in breast cancer development, miRNAs have also been shown to oscillate with the circadian cycle and target circadian-relevant genes. In *Arabidopsis*, several miRNAs were shown to oscillate based on environmental light, exhibiting higher levels during the daytime than during the nighttime, and oscillation on the basis of photic control and not an internal clock [10]. In a study conducted on HeLa cell lines, results showed that the miR-192/194 cluster directly regulates the entire *Period* gene family, with increased expression of the miR192/194 tandem causing lowered expression of the *Period* genes and a shortening of the circadian cycle [11]. Further investigation has illustrated that miR-132 expression is induced by light within the suprachiasmatic nucleus (SCN) and that it has the capacity to restore homeostasis and reset the activity caused to the circadian clock by nocturnal light [12]. Interestingly, a recent study has also reported that miR-132 levels are lowered in breast cancer cells, and identified miR-132 as a tumor suppressor that acts by inhibiting proliferation, migration, invasion, and metastasis [13].

Despite the increasing evidence that miRNAs play a role in breast cancer development and in circadian rhythms and that these roles likely overlap, information on the involvement of miRNAs in CD-induced breast cancer remains scarce. In the handful of studies that have investigated epigenetic modifications in CD-induced breast cancer, all the experiments have focused on changes in DNA methylation [14-16]. Although the most recent study of epigenetic CD-induced breast cancer conducted a DNA methylation analysis on shift worker blood samples at miRNA promoters and identified increased methylation of breast cancer relevant miRNAs, the study did not measure expression levels of miRNAs or investigate CD-induced changes in mammary tissues [17, 18]. Furthermore, although the DNA methylation studies mentioned have provided valuable insights into the epigenetic role in CD-induced breast cancer, none of them have investigated the effect of varying degrees of CD or the influence of fluctuations within a 24-hour circadian cycle. Therefore, to shed new light on the epigenetic mechanisms involved in CD-induced breast cancer, the current study utilized a photoperiod-shifting (PS) paradigm involving various degrees of CD and specific time points within a circadian cycle to investigate the influence of CD on miRNA expression in the mammary tissues of a rodent model system.

## RESULTS

### Circadian disruption causes aberrant expression of a broad range of miRNAs, and the changes are linked to an early time point in the circadian cycle

In this study, we investigated the effect of varying degrees of CD on miRNA expression in the mammary tissues of Sprague Dawley rats. The influence of light-dependent zeitgeber times (ZT) was also incorporated to investigate possible fluctuations within a 24-hour circadian cycle. The sequencing results identified a broad range of miRNAs that were differentially expressed between the circadian-disrupted samples and the control samples (Table 1). Amongst the differentially expressed miRNAs, all but one play a role in breast cancer development (Table 1). Numerous miRNAs that oscillate with the circadian cycle and/or have predicted circadian-relevant targets were also differentially expressed due to circadian disruption (Table 1).

**Figure 1:**
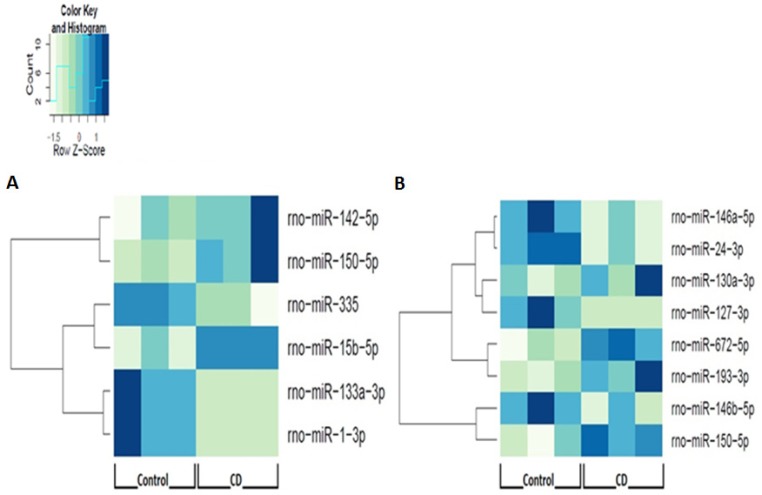
Circadian disruption causes changes in miRNA expression in both the acute and chronic groups Heat map dendrograms based on the Illumina sequencing results for the differentially expressed miRNAs in the **A.** 24-hour acute ZT06 group and **B.** two-week chronic ZT06 group. All the differentially expressed miRNAs represent a significant expression difference, *p*-adjusted < 0.1, *N* = 6.

**Figure 2:**
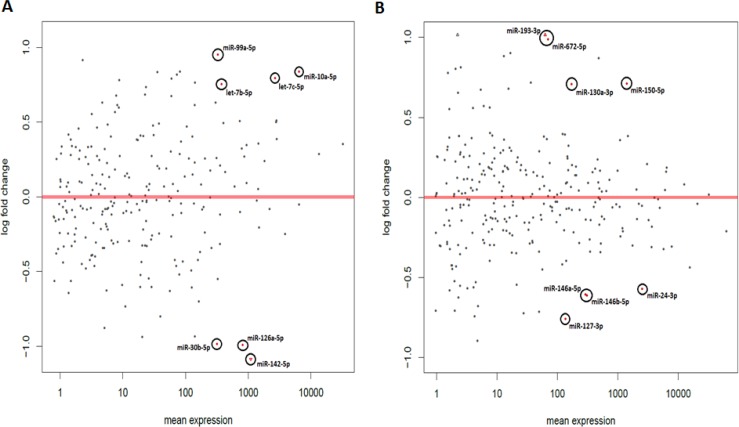
Chronic circadian disruption causes significant changes in miRNA expression in the ZT06 groups MA plot based on the small RNA Illumina sequencing results for the **A.** 24-hour chronic ZT06 group and **B.** two-week chronic ZT06 group. The y-axis represents log fold change, while the x-axis represents mean expression. The plots represent all the miRNA expressions that were identified, and the red plots represent miRNAs that showed a significant expression difference; *p*-adjusted < 0.1, *N* = 6. Each red plot is identified with the corresponding miRNA.

**Figure 3:**
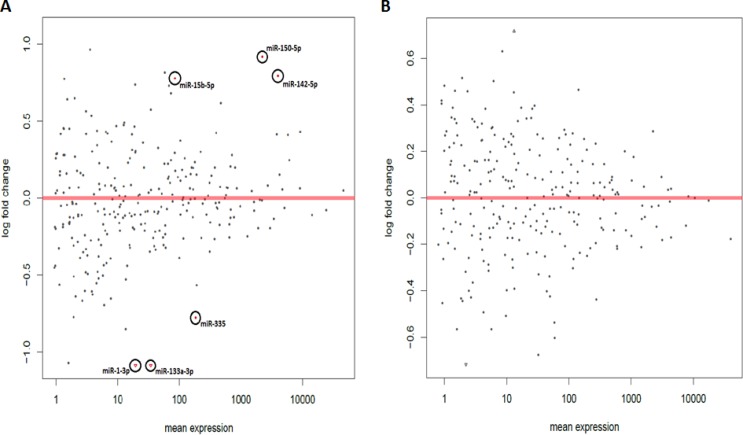
Acute circadian disruption causes significant changes in miRNA expression in the 24-hour acute ZT06 group MA plot based on the small RNA Illumina sequencing results for the **A.** 24-hour acute ZT06 group and **B.** two-week acute ZT06 group. The y-axis represents log fold change, while the x-axis represents mean expression. The plots represent all the miRNA expressions that were identified, and the red plots represent miRNAs that showed a significant expression difference; *p*-adjusted < 0.1, *N* = 6. Each red plot is identified with the corresponding miRNA.

**Figure 4:**
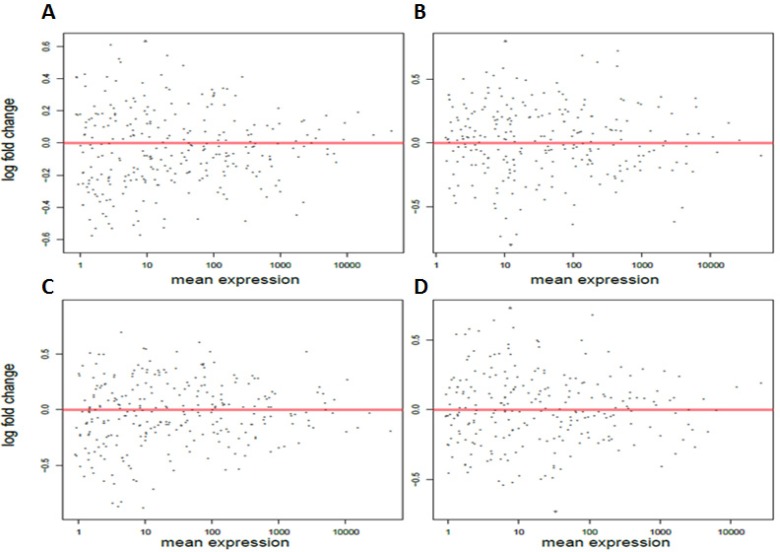
Chronic and acute circadian disruption causes no significant changes in miRNA expression in all the ZT19 groups MA plots based on the small RNA Illumina sequencing results for the **A.** 24-hour chronic ZT19 group, **B.** two-week chronic ZT19 group, **C.** 24-hour acute ZT19 group, and **D.** two-week acute ZT19 group. The y-axis represents log fold change, while the x-axis represents mean expression. The plots represent all the miRNA expressions that were identified. None of the miRNAs in the ZT19 groups illustrated significant expression differences.

The changes in miRNA expression were observed in both the acute and chronic circadian-disrupted groups, as well as in both the 24-hour and two-week tissue-extraction groups (Figures 1–3). Only the two-week acute group did not show any changes in miRNA expression (Figures 3 and 4). Interestingly, the time of tissue extraction based on ZT influenced the results. None of the ZT19 groups showed any changes in miRNA expression (Figure 4), whereas with the exception of the two-week acute group, all the ZT06 groups showed differences in miRNA expression compared to the respective control groups (Figures 2 and 3). The ZT19 and ZT06 extraction time points represented the dark and light phases of the circadian cycle, respectively, indicating that the miRNA expressions correlated to light-dependent time points within the circadian cycle.

Between the ZT06 groups that showed differences in miRNAs, there were no significant correlations or patterns in specific miRNA expression, with only two miRNAs being differentially expressed in more than one group, miR-150-5p and miR-142-5p (Table 2). Furthermore, with the exception of no differential expression in the two-week acute ZT06 group, there was no significant difference in the number of differentially expressed miRNAs based on the degree of CD (Table 2).

In terms of expression patterns within each individual ZT06 group, the 24-hour acute group had a tandem cluster that was differentially expressed, with downregulation of the tumour suppressor miRNAs, miR-1 and miR-133a (Tables 1 and 2). Within the 24-hour chronic ZT06 group, two miRNAs from the same gene family that are linked to breast cancer and circadian rhythms, let-7b and let-7c, were differentially expressed (Tables 1 and 2). Finally, in the two-week chronic ZT06 group, both miR-146a and miR-146b, which belong to the same gene family, were downregulated (Tables 1 & 2).

**Table 1:** Circadian disruption induces expression changes in a broad range of breast cancer relevant miRNAs Differentially expressed miRNAs based on Illumina sequencing in all the circadian-disrupted samples and their links to breast cancer development and circadian rhythms.

miRNA	Links to Breast Cancer Development	Links to Circadian Rhythms
let-7b-5p	Tumour suppressor linked to cell motility in breast cancer	Predicted target Cry2; Circadian oscillation
let-7c-5p	Tumour suppressor in breast cancer	Predicted target Cry2
miR-1-3p	Tumour suppressor in various cancers, possibly linked to BC	Predicted target Clock
miR-10a-5p	Amplified in and linked to ER+ breast cancer	
miR-15b-5p	Tumour suppressor in BC linked to BCL2 expression	
miR-24-3p	Enhances tumour invasion and metastasis	Predicted target Per2
miR-30b-5p	Linked to tumourigenesis and metastasis in breast cancer	
miR-99a-5p	Tumour suppressor in BC linked to mTOR expression	Circadian oscillation
miR-126a-5p	Linked to metastasis in breast cancer	Predicted target NPAS2
miR-127-3p	Tumour suppressor in BC linked to BCL6 expression	
miR-130a-3p	OncomiR in breast cancer	
miR-133a-3p	Tumour suppressor in BC linked to tumour growth and invasion	Predicted target TIMELESS
miR-142-5p	Linked to ER/PR status in breast cancer	Circadian oscillation
miR-146a-5p	Tumour suppressor in breast cancer linked to NFκB	Circadian oscillation
miR-146b-5p	Tumour suppressor in breast cancer linked to STAT3 and NFκB	Predicted target TIMELESS; Circadian oscillation
miR-150-5p	OncomiR in breast cancer linked to apoptosis	Circadian oscillation
miR-193-3p	Tumour suppressor in breast cancer	Predicted target Cry2
miR-335	Linked to breast cancer development and BRCA1	Predicted target Clock
miR-672-5p	----------------------------------	

**Table 2:** Circadian disruption induces potentially aberrant miRNA expression patterns Expression patterns of all the differentially expressed miRNAs based on Illumina sequencing in the circadian-disrupted groups. Only the ZT specific groups that showed changes in miRNA expression are depicted. Colour coordination represents miRNAs that are part of the same gene family, cluster, or the same miRNA.

24-Hour Acute ZT06	Expression	24-Hour Chronic ZT06	Expression	2-Week chronic ZT06	Expression
rno-miR-142-5p	Over	rno-miR-126a-5p	Under	rno-miR-146a-5p	Under
rno-miR-150-5p	Over	rno-miR-30b-5p	Under	rno-miR-24-3p	Under
rno-miR-335	Under	rno-let-7b-5p	Over	rno-miR-130a-3p	Over
rno-miR-15b-5p	Over	rno-miR-99a-5p	Over	rno-miR-127-3p	Under
rno-miR-133a-3p	Under	rno-miR-10a-5p	Over	rno-miR-672-5p	Over
rno-miR-l-3p	Under	rno-let-7c-5p	Over	rno-miR-193-3p	Over
		rno-miR-142-5p	Under	rno-miR-146b-5p	Under
				rno-miR-150-5p	Over

### Validation of sequencing results through qRT-PCR verifies miR-127 expression

**Figure 5:**
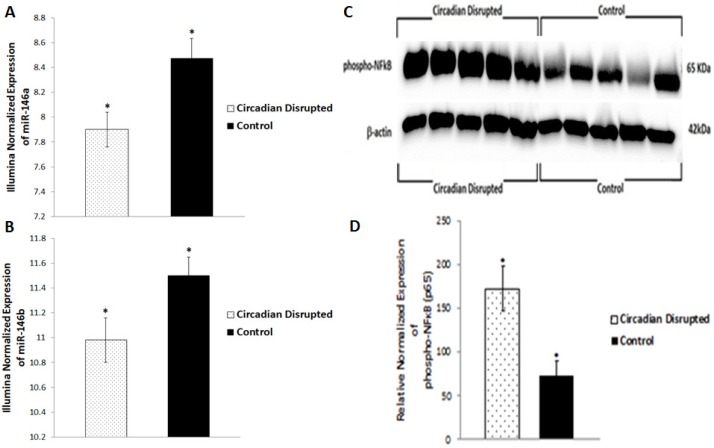
Circadian disruption causes lowered expression of miRNAs 146a and 146b, and increased expression of activated NF-κB **A.** Mean relative expression of miR-146a based on Illumina sequencing, *p-adjusted < 0.1, *N* = 6. **B.** Mean relative expression of miR-146b based on Illumina sequencing, *p-adjusted < 0.1, *N* = 6. **C.** Western immunoblotting images of phospho-NFκB (p65) and β-actin from a 6% SDS-PAGE gel for the two-week chronic ZT06 group. Images were taken with the FluorChem HD2. The five samples on the left represent the control samples, and the five samples on the right represent the circadian disrupted samples. **D.** Mean relative expression of the phospho-NFκB (p65) protein based on the actin band in C, *p < 0.05, *N* = 10. Error bars represent SEM.

**Figure 6:**
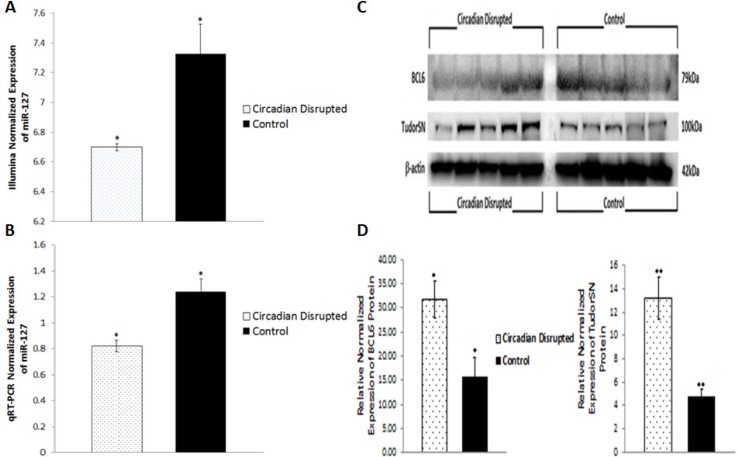
Circadian disruption causes decreased expression of miR-127, and increased expression of Tudor-SN and BCL6 **A.** Mean relative expression of miR-127 based on Illumina sequencing, *p-adjusted < 0.1, *N* = 6. **B.** Mean Relative expression of miR-127 based on qRT-PCR data, *p < 0.05, *N* = 10. **C.** Western immunoblotting images of BCL6, Tudor-SN, and β-actin from a 10% SDS-PAGE gel for the 2-week chronic ZT06 group. Images were taken with the FluorChem HD2. The five samples on the left represent the circadian disrupted samples, and the five samples on the right represent the control samples. **D.** Mean relative expression of the BCL6 and Tudor-SN proteins based on the actin band in C,*P < 0.05, *N* =10.

Of the three ZT06 groups that illustrated differential expression of miRNAs due to CD, emphasis was placed on the two-week chronic ZT06 group due to the differential expression of miRs 146a and 146b, and miR-127 (Figures 5A-5B and 6A). Confirmation of the two-week chronic ZT06 miRNA sequencing results was attempted through small RNA qRT-PCRs. Unfortunately, the biggest log fold change in miRNA expression based on Illumina sequencing in this group was approximately 1 (Figure 2). This made verification of the sequencing results difficult, both because of the sensitivity of qRT-PCR and the fact that qPCR is not an infallible validation method, especially when the technology being validated incorporates PCR-based amplification, as does Illumina [19]. Amongst the qRT-PCRs performed on the differentially expressed miRNAs in the two-week chronic ZT06 group, the miR-127 qPCR results validated the sequencing results. The qPCR data illustrated a similar and consistent change in relative miR-127 expression when compared to the Illumina sequencing (Figure 6A and 6B).

### Circadian-disruption-induced changes in miRNA expression in the two-week chronic ZT06 group correlated to aberrant levels of breast-cancer-relevant proteins

**Figure 7:**
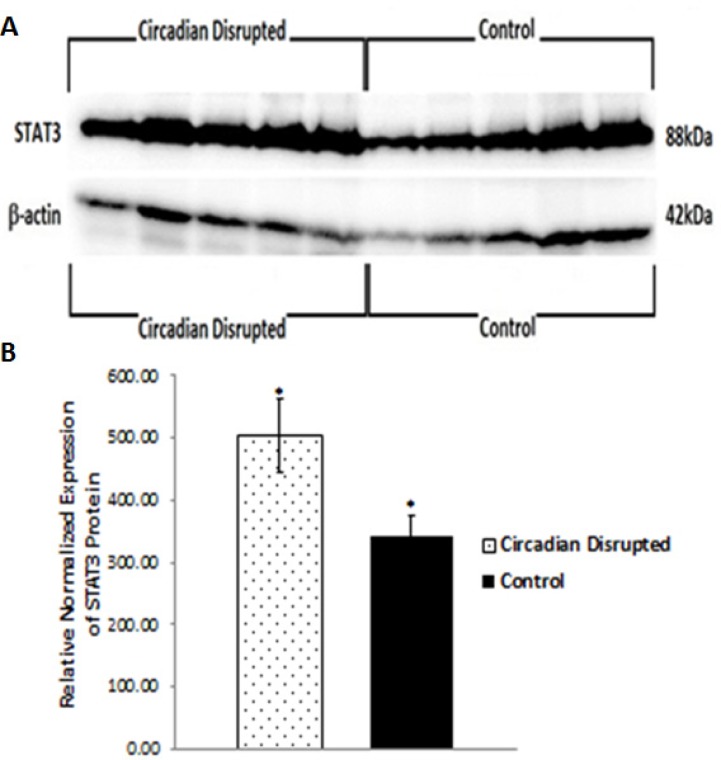
Circadian disruption causes increased expression of STAT3 **A.** Western immunoblotting images of STAT3 and β-actin from a 10% SDS-PAGE gel for the two-week chronic ZT06 group. Images were taken with the FluorChem HD2. The five samples on the left represent the circadian disrupted samples, and the five samples on the right represent the control samples. **B.** Mean relative expression of the STAT3 protein based on western immunoblotting data for the two-week chronic ZT06 group. Expressions were normalized to the actin band in A, *p < 0.05, *N* = 10. Error bars represent SEM.

**Figure 8:**
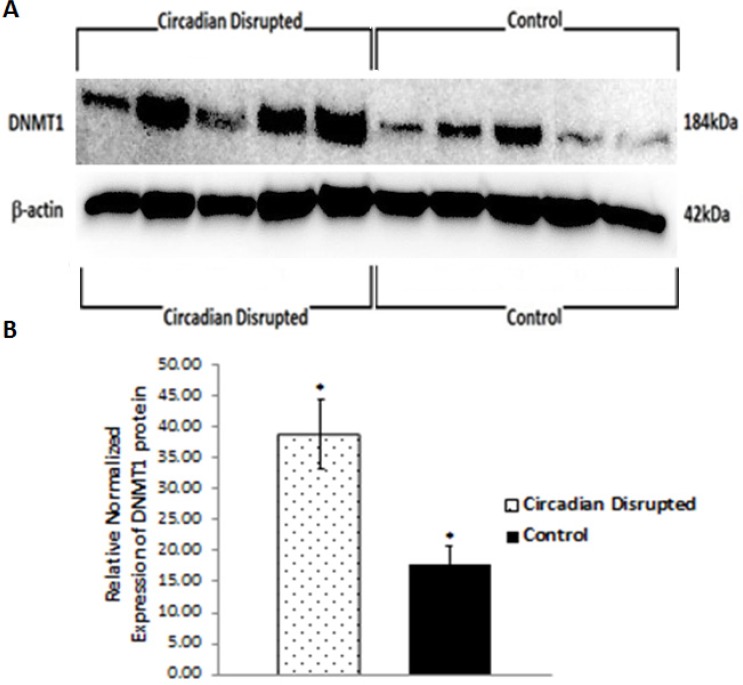
Circadian disruption causes increased expression of DNMT1 **A.** Western immunoblotting images of DNMT1 and β-actin from a 6% SDS-PAGE gel for the two-week chronic ZT06 group. Images were taken with the FluorChem HD2. The five samples on the left represent the control samples, and the five samples on the right represent the circadian disrupted samples. **B.** Mean relative expression of the DNMT1 protein based on western immunoblotting data for the two-week chronic ZT06 group. Expressions were normalized to the actin band in A, *p < 0.05, *N* = 10. Error bars represent SEM.

To investigate the potential downstream consequences of changes in miRNA expression due to CD in the two-week chronic ZT06 group, western blot analysis was performed on relevant proteins linked to the differentially expressed miRNAs and breast cancer development. With respect to miR-146a and 146b activity, the results illustrated higher quantities of the transcriptionally active form of NF-kappaB (Figure 5). Phospho-NFκB (p65) protein levels showed a nearly 2.5-fold increase in the circadian-disrupted samples compared to the control samples, and a p-value of less than 0.05 (Figure 5D). The results also illustrated higher quantities of a verified target of miR-127 and a protein linked to cellular senescence, BCL6 (Figure 6). BCL6 protein levels showed a twofold increase in the circadian-disrupted samples, and a p-value of less than 0.05 (Figure 6D). Western blot analysis also showed higher quantities of Tudor-SN, a protein linked to NF-kappaB activity and lower miR-127 expression (Figure 6). Tudor-SN protein levels showed an almost threefold increase in the circadian-disrupted samples compared to the control samples and a p-value of less than 0.01 (Figure 6D). Western blot analysis was also conducted on STAT3 (Figure 7). The data showed that STAT3 protein levels had a 0.5-fold increase in the circadian disrupted samples compared to the control samples, and a p-value of less than 0.05 (Figure 7B). Increased STAT3 levels are linked to lower expression of miR-146b and increased NF-κB activity, both of which were present in the two-week chronic ZT06 group (Figure 5). To investigate whether DNA methylation differences may have contributed to the aberrant expression of the differentially expressed miRNAs, western blot analysis was conducted on the maintenance DNA methyltransferase, DNMT1 (Figure 8). The results showed that DNMT1 protein levels increased more than twofold in the circadian-disrupted samples compared to the control samples, with a p-value of less than 0.05 (Figure 8B).

## DISCUSSION

Knowledge on the influence of varying degrees of CD and miRNA activity in CD-induced breast cancer remains scarce. Our study, which induced CD using a photoperiod-shifting paradigm shown to cause behavioural and physiological changes in rodents, is to our knowledge the first to illustrate that CD induces changes in miRNA expression in rodent mammary tissues [20-23]. Both the acute and chronic CD schemes employed resulted in the differential expression of a variety of breast-cancer-relevant and potentially circadian-relevant miRNAs (Table 1 and Figure 1). The 54 day chronic CD scheme, caused changes in miRNA expression in both the 24-hour and two-week chronic extraction groups (Figure 2), indicating that long-term CD can have aberrant consequences on breast cancer development through miRNA activity. Because the 6 day acute CD scheme, also caused aberrant changes in miRNA expression in the 24-hour group (Figure 3), this indicates that even short-term CD may induce potentially harmful changes that can increase the risk of circadian-related diseases.

Amongst the experimental groups in the study, only the two-week acute group did not show any significant changes in miRNA expression (Figures 3 and 4). The two-week acute group underwent the least rigorous CD-scheme and had the longest reentrainment period before tissue extraction. This information, coupled with the fact that the 24-hour acute and two-week chronic groups did exhibit changes in miRNA expression (Figure 1), indicates that the CD-induced changes in miRNA levels may be linked to the degree of CD and a correlated length of reentrainment to restore normal miRNA expression. These results are supported by evidence from previous studies indicating that CD effects on various processes can be plastic. By utilizing the same acute photoperiod-shifting paradigm used in the present study, a study used behavioural measurements to show that rats required 17 days of a 12-12 light-dark cycle to attain reentrainment [22]. In terms of epigenetic modifications, a study illustrated that CD-induced changes in DNA methylation can be reversed, showing that circadian disrupted mice that are entrained back to a regular 24-hour day exhibit a reversion in DNA methylation levels [24]. Based on our results, it would seem that 14 days or fewer on a 12-12 light-dark cycle is enough time to cause a reversion in miRNA levels due to the acute CD scheme used, but not enough time to cause reversion due to the chronic CD scheme. Along with evidence from previous literature, this indicates that CD-induced miRNA changes are likely plastic, with varying degrees of CD requiring varying lengths of reentrainment to revert the changes in miRNA expression.

Through the incorporation of light-dependent ZT extractions, our results also illustrate that specific time points within a circadian cycle may play an important role in miRNA activity and CD-induced breast cancer. Specifically, the results showed that none of the groups representing the dark phase of the circadian cycle, the ZT19 extraction groups, exhibited any changes in miRNA expression (Figure 4). In terms of the ZT06 groups, which represent the light phase of the circadian cycle, all but the two-week acute group showed differences in miRNA levels (Figures 2 and 3). Previous literature has demonstrated that miRNAs can oscillate throughout a 24-hour circadian cycle. In particular, a microarray-based experiment on mouse liver revealed that over 13% of the probed miRNAs exhibited circadian expression patterns [25]. Interestingly, some of the miRNAs identified in that study are miRNAs that exhibited differential expression in our study (Table 1). Therefore, our findings further stress that specific time points within a 24-hour circadian cycle are important for accurately measuring miRNA levels and activity, and that these details need to be considered when investigating circadian-relevant diseases. For example, melatonin, a pineal hormone that is linked to circadian rhythms and exhibits fluctuations in its daytime and nocturnal concentrations, has been shown to influence human breast cancer xenografts through a day-night rhythm of tumour proliferation, fatty acid uptake, metabolism, and signal transduction activity [26, 27]. Research has also reported that DNA methylation activity may be influenced by light-dependent induction or repression of DNA methylation enzymes [24]. Therefore, given these previous findings, coupled with the fact that miRNAs can influence cancer-relevant processes and also fluctuate with the circadian cycle, it is not inconceivable that circadian-dependent fluctuations in miRNAs can influence breast cancer development. Our results illustrate a clear pattern of CD-induced changes in miRNA expression based on the light and dark phases of the circadian cycle (Figures 2–4), and all but one of the differentially expressed miRNAs are linked to breast cancer development (Table 1). It is therefore likely that circadian fluctuations in miRNA activity are an important component in the development of CD-induced breast cancer and should not be ignored.

In terms of specific miRNAs that were differentially expressed due to CD, all but one have been linked to breast cancer development, with many of the CD-induced expressions correlating to potentially aberrant consequences (Tables 1 and 2). Although only two miRNAs were identified in more than one of the ZT06 groups, miR-142-5p and miR150-5p (Table 2), there are significant correlations and patterns related to breast cancer development in each ZT06 group. In the 24-hour acute ZT06 group, two miRNAs that are part of the same cluster, miR-133a and miR-1, were both underexpressed (Table 2). The miR-1/133a cluster has been shown to be downregulated in a variety of cancers, whereas miRNA-133a has also been shown to act as a tumour suppressor in breast cancer cells by causing S/G_2_ phase cell-cycle arrest through activity on phosphorylated Akt [28, 29]. Furthermore, two predicted circadian-relevant targets of the miR 1/133a cluster are CLOCK and TIMELESS (Table 1). Previous literature has shown that CLOCK knockdown in breast cancer cells results in increased expression of various tumour suppressor genes and decreased expression of multiple oncogenes, indicating an oncogenic influence by CLOCK that is potentially an early event in breast cancer development [30]. TIMELESS, a circadian and cell-cycle regulator that may act as a molecular bridge between these two regulatory systems, has been reported to be overexpressed in many breast cancer cells, with the increased expression being linked to increased proliferation and poorer prognostic outcome [31, 32]. This indicates that CD-induced down regulation of the miR-1/133a cluster may have oncogenic effects through previously identified mechanisms, as well as through potential circadian relevant targets.

In the two-week chronic ZT06 group, two miRNAs from the same gene family, miR-146a and 146b, were downregulated (Table 2). The expression of miR-146a and 146b has been shown to suppress NF-κB activity [33, 34]. A key component of inflammation and innate immunity, NF-κB is a protein complex that controls the transcription of numerous genes and has been implicated as a key component in many steps of cancer initiation and progression [35-37]. Activation of NF-κB can result in the upregulation of anti-apoptotic genes and contribution to cell survival and proliferation [36]. In breast cancer, NF-KappaB has been shown to increase cell migration and promote tumor-initiating cells [35, 38]. Our western blot results show that activated NF-κB was significantly increased in the circadian-disrupted samples of the two-week chronic ZT06 group (Figure 5C-5D). This finding is consistent with a previous study that indirectly correlated increased induction of NF-κB pathways to CD-induced promoter methylation of miR-219 [18]. The present study shows that CD results in a direct increase of NF-κB activity in mammary tissues and that this activity may be linked to CD-induced downregulation of miR-146a and 146b (Figure 5).

Cellular senescence is a response to extracellular and intracellular stresses, such as oncogenic stimuli, that causes permanent cell-cycle arrest and acts as a potent tumour suppression mechanism that prevents the oncogenic transformation of primary cells [39]. miRNA-127-3p has been shown to be upregulated in senescent cells, and its downregulation has been shown to promote cell proliferation [40, 41]. Small RNA sequencing and validation through qRT-PCR showed that miR-127 is underexpressed in the two-week chronic ZT06 group (Figure 6A-6B). Because cell senescence plays an important role in tumour initiation, this dynamic warranted further investigation [42]. Amongst the proteins linked to miR-127 activity and breast cancer development, are BCL6 and Tudor-SN. A target of miR-127, the BCL6 gene has been identified as a potent inhibitor of cell senescence and a contributor to oncogenesis, and the western blot data showed that BCL6 levels were significantly increased in the circadian-disrupted samples (Figure 6) [43, 44]. Tudor-SN is a member of the RISC complex and also known as SND1, and it has been implicated in various cancers and has been shown to regulate miRNA processing and expression by degrading primary-miRNA transcripts that undergo adenosine deaminase acting on RNA (ADAR) editing [45-48]. In terms of its link to cancer progression, Tudor-SN has been shown to promote resistance to apoptosis and to be induced by NF-κB, which as mentioned above, was increased in the CD-induced tissues through potentially decreased miR-146a and 146b expression (Figure 5) [45, 49]. Amongst the miRNAs influenced by Tudor-SN, is miR-127, and studies have shown that Tudor-SN triggers miR-127 downregulation in breast cancer cells [50]. Our western blot results illustrate that Tudor-SN was significantly increased in the CD-induced tissues (Figure 6). This indicates that CD causes aberrant expression of Tudor-SN, which may be linked to increased NF-κβ activity through CD-induced repression of miR-146a and 146b. Consequently, this dynamic potentially results in the decreased expression of miR-127 through increased Tudor-SN activity, which in turn results in increased expression of the proto-oncogene BCL6.

Numerous publications have provided evidence that the STAT3 gene produces oncogenic effects in a variety of cancers, including being active in 50–60% of primary breast tumours and being linked to the promotion of breast cancer stem cell traits [51, 52]. Our western blot data illustrate that STAT3 levels showed a significant increase in the CD-induced samples in the two-week chronic ZT06 group (Figure 7). Interestingly, STAT3 has also been identified as part of a negative feedback loop with miR-146b. In healthy cells, the gene encoding miR-146b is a direct target of STAT3, and as STAT3 levels increase, it results in the activation of miR-146b [34, 53]. The activation of miR-146b results in the attenuation of NF-κB activity, which in turn, results in the subsequent inhibition of STAT3 expression [34, 53]. In breast cancer cells, increased promoter methylation of miR-146b results in inhibition of the negative feedback loop and increased expression of STAT3 [53]. As mentioned, miRNA146b levels were downregulated in the circadian-disrupted samples and NF-κB activity was also increased (Figure 5). Although no promoter-specific DNA methylation analysis was performed in this study, western blot data indicate that there were increased levels of DNMT1 in the circadian-disrupted samples (Figure 8). Together with previous literature that has shown that CD results in increased miRNA promoter methylation, this indicates that the aberrant NF-κB activity and STAT3 levels could potentially be a consequence of miR-146b promoter methylation due to increased levels of DNMT1 [18, 54]. Furthermore, the STAT3 protein has also been shown to induce BCL6 expression by binding to one of two regulatory regions within the BCL6 gene [55]. As a result, the CD-induced increases in STAT3 expression through NF-κB activity may not only cause oncogenic effects on their own, but also contribute to the increase in BCL6 levels. In view of the previously discussed results, it seems that CD results in a cascade of interrelated activity that revolves around increased NF-κB activity and expression of BCL6, with aberrant CD-induced downregulation of miR-127 and miR-146b potentially contributing to this cascade.

Our findings illustrate for the first time that CD induces changes in miRNA levels in mammary tissues. In addition, these CD-induced changes are likely plastic and potentially linked to the light and dark phases of the circadian cycle. Amongst the differentially expressed miRNAs reported, are breast-cancer-relevant miRNAs that also have predicted circadian-relevant targets linked to breast cancer development. The basis of the link between miRNA activity and CD-induced breast cancer seems to be an interconnected web of increased NF-κB activity and BCL6 expression, likely linked to and promoted by the CD-induced decrease in miR-127 and miR-146b. Because inflammation, innate immunity, and cellular senescence are crucial in tumour initiation and breast cancer progression, and because these two miRNAs have been linked to these mechanisms, it is likely that all of them are key, interrelated components in the potential initiation and development of CD-induced breast cancer. Although our findings are supported by previous literature, given that they represent the first evidence of potentially direct consequences of aberrant CD-induced miRNA activity in mammary tissues, further investigation is required. A stricter CD scheme in terms of ZT extractions and extractions following CD is warranted for developing a more accurate range of CD-induced miRNA plasticity and for providing more details on the potential consequences of light-dependent miRNA fluctuations in CD-induced breast cancer. Verification of potential circadian-relevant targets through luciferase reporter experiments, incorporation of the CD schemes into xenograft models, and further investigation of the dynamic of miR-127 and miR-146b activity in cancer cells are all logical extensions of the present study. In either case, whatever the next avenue may be, this study provides evidence that CD induces changes in miRNA levels in mammary tissues with potentially malignant consequences, thus indicating that the role of miRNAs in CD-induced breast cancer should not be dismissed.

## MATERIALS AND METHODS

### Animal model and circadian-disruption paradigm

Female Sprague Dawley rats from Charles River (Quebec) were housed at the Canadian Center for Behavioural Neuroscience at the University of Lethbridge. The rats were housed in a sterile facility in a temperature-controlled room, two per cage, and given food and water *ad libitum*. Handling and care of the animals was performed in accordance with the recommendations of the Canadian Council on Animal Care, and procedures were approved by the University of Lethbridge Animal Welfare Committee. Before the start of the experiment, all the rats were entrained to a 12-hour light-dark cycle for 22 days to allow entrainment to a normal light schedule. At 83 days old, the rats were then randomly assigned to different treatment and control groups.

CD was induced by following a photoperiod-shifting paradigm that has been shown to cause physiological and behavioural changes in rodents [20-23]. In total, 40 female rats underwent this PS paradigm. To stimulate PS, the colony lights were turned on three hours earlier on each successive day of the experiment. To investigate the effect of varying degrees of CD, the 40 rats were separated into acute and chronic CD groups. Twenty rats underwent acute photoperiod shifting, which consisted of lights coming on three hours earlier each day for a total cycle time of six days (Supplementary Table S1). Another 20 rats underwent chronic photoperiod shifting, which consisted of a rotation between a six-day period in which lights came on three hours earlier each day and a 10 day period in which a regular 12-hour light-dark cycle was followed, for a total cycle time of 54 days (Supplementary Table S2). For both the acute (six days) and chronic groups (54 days), following the PS cycle, the rats were exposed to a normal 12-hour light-dark cycle until it was time for tissue extraction.

The acute (20 rats) and chronic (20 rats) CD groups were then separated further based on the time of tissue extraction (Supplementary Figure S1). Mammary tissue extractions occurred 24 hours and two weeks following acute or chronic CD, with 10 rats from each CD group undergoing tissue extraction at each of these times. To account for and investigate the potential influence of specific time points within a 24-hour circadian cycle, two different tissue-extraction time points, each corresponding to a specific ZT, were chosen for each tissue-extraction day (24 hours and two weeks following CD). Half of the rats (five) in each tissue-extraction group were sacrificed at ZT06 (six hours after lights on), and the remaining five rats in each group were sacrificed at ZT19 (19 hours after lights on). These two ZT points were chosen because they represented the light and dark phases of the circadian cycle.

Both the acute (20 rats) and chronic (20) control groups were exposed to a 12-hour light-dark cycle for either six days (acute) or 54 days (chronic). The rats from each CD control group were then exposed to a 12-hour light-dark cycle for either 24 hours (10 rats) or two weeks (10 rats), depending on the time of tissue extraction for the corresponding experimental group. From each tissue-extraction control group (10 rats), five rats were sacrificed at ZT06 and five rats at ZT19 on the corresponding tissue-extraction day.

Euthanasia of the rats was performed through anaesthesia with Isoflurane (4–5%; oxygen at 2 litres per minute) and decapitation by a guillotine, with euthanasia of the rats alternating between the control and experimental rats. The mammary glands were collected, immediately stored in liquid nitrogen, and stored long-term at −80°C.

### Total RNA extraction

Whole mammary tissues were ground in liquid nitrogen using sterile, chilled mortars and pestles. Approximately 0.05 g of ground tissue from each sample was then suspended in Zymo Research tri-reagent (R2053) and lysed using two cycles of the Qiagen Tissue Lyser II for 2 minutes at 25 Hz. Total RNA was extracted using the direct-zol RNA Miniprep kit from Zymo Research (R2053). The quality of the RNA was then checked using NanoDrop 2000c, and quality bioanalysis was conducted using the Agilent 2100 and the Agilent Small RNA Kit and Chip (5067-1548); only samples with an RNA integrity value (RIN) greater than 8 were used in downstream applications.

### Small RNA sequencing and bioinformatics analysis

Three samples from each tissue-extraction and ZT group for both the experimental and control groups were randomly chosen to undergo sequencing analysis. The TruSeq Small RNA Sample Preparation Kit for 25–36 indexes from Illumina (RS-200-0036) was used to prepare small RNA libraries from 1 μg of total RNA. Six-percent SDS-PAGE gels were run to cut out the bands of interest for library validation on the Agilent 2100 using the Agilent High Sensitivity DNA Kit and Chip (5067-4626). Cluster generation for sequencing was performed using cBot and the TruSeq SR Cluster Kit v2-cBot-GA (GD-300-2001) from Illumina. Single-end sequencing was performed using the TruSeq SBS Kit v5-GA (FC-104-5001) from Illumina on the Genome Analyzer GAIIx at 36 cycles.

Bioinformatics analysis was performed on the sequencing data to define expression levels of miRNAs amongst the different experimental groups. Quality of the libraries was evaluated using FastQC v0.10.1 software. Adapter trimming was done using Cutadapt (https://code.google.com/p/cutadapt/). Trimmed reads were converted to fasta using an ad-hoc perl script and collapsed to unique tags with the fastx_collapser program from the FASTX-Toolkit (http://hannonlab.cshl.edu/fastx_toolkit/). Unique tags in fasta format were aligned to mature miRNA sequences downloaded from miRBase (release 19) using Micro-Razers; only unambiguous alignments were kept [56]. An ad hoc perl script was used to process the MicroRazers alignment file to produce a tab delimited count file containing miRNA IDs and raw read count columns.

### Small RNA qRT-PCR

Validation of the sequencing results was performed by qRT-PCR. Small RNA cDNA was synthesized from 150 ng of RNA using the reagents and protocol associated with the GeneCopoeia All-in-One miRNA First-Strand cDNA Synthesis Kit (AMRT-0060). The small RNA qPCRs were performed utilizing SYBR Green on the BioRad C1000 Thermal Cycler and CFX96 Real-Time System, by using the reagents and protocol associated with the GeneCopoeia All-in-One miRNA qRT-PCR detection kit (AOMD-Q060). All reactions were run in triplicate, and the program used was the one recommended by GeneCopoeia (10 min at 95°C; 40 cycles of 10 sec at 95°C, 20 sec at 60°C, and 10 sec at 72°C). Efficiency standard curves for the primers were generated using serial dilutions, and following all the qPCR cycling, melt curve analysis was conducted using the optimal parameters for the BioRad C1000 thermal cycler (65°C to 95°C, increments of 0.5°C).

The reverse primer used for all the reactions was the Universal Adaptor Primer included in the miRNA qRT-PCR detection kit from GeneCopoeia (AOMD-Q060). All the forward primers were miRNA-specific primers ordered from GeneCopoeia based on the target of interest, for example, rno-miR-127-3p (RmiRQP0111). Based on previous publications, recommended endogenous reference genes were ordered from GeneCopoeia and tested for stability [57, 58]. The best combination of two reference genes, rno-miR16 (RmiRQP0227) and rno-let-7a (RmiRQP0002), was found using the programs NormFinder (http://moma.dk/normfinder-software) and qbase^plus^ (Biogazelle), with the stability values meeting the geNorm stability cutoffs (CV < 0.25, M-Value < 0.5). These two reference genes have been recommended as control genes for miRNA expression analysis in breast cancer [57].

### Western blot analysis

A 0.07 g portion of each mammary tissue sample was lysed and sonicated in 350 ul of 1%SDS+ProteaseInhibitor. Next, the samples were placed in a 95°C water bath for 5 minutes and then centrifuged at 10,000 g for 10 minutes at 4°C. The supernatant was collected and centrifuged at 10,000g for 10 minutes at 4°C. Protein content was determined with the Bradford protein determination assay from BioRad. Equal amounts of lysate protein (10 ug/10 uL) were then run on 6–10% SDS-polyacrylamide gels and transferred to PVDF membranes from GE Healthcare.

Western immunoblotting was conducted following well established protocols [59]. Unaltered PVDF membranes were stained with Coomassie Blue (BioRad) to ensure even blotting of the proteins. Membranes were incubated with various primary antibodies at different dilutions based on the antibody company, antibodies against rabbit anti-BCL6 (1:1000, Abcam), mouse anti-Tudor-SN (1:500, Santa Cruz), rabbit anti-STAT3 (1:500, Santa Cruz), rabbit anti-phospho-NFκB p65 (Ser311) (1:500, Santa Cruz), rabbit anti-DNMT1 (1:500, Santa Cruz), and mouse anti-βactin (1:1000, Abcam). Antibody binding was revealed by incubation with horseradish peroxidase-conjugated secondary antibodies based on the primary antibody source animal (Santa Cruz) and the ECL Plus immunoblotting detection system (GE Healthcare). Chemiluminescence was detected using the FluorChem HD2 from Cell Biosciences. Signals were quantified using NIH ImageJ software and normalized to the actin protein band.

### Statistical analyses

For statistical analyses of the bioinformatics data, raw count files were loaded into R version 3.1.2. All the statistical comparisons were performed using the DESeq2 (version 1.4.5) bioconductor package as described in the package manual [60]. Multiple comparisons adjustment was performed using Benjamini-Hochberg procedure, and microRNAs with an adjusted p-value below 0.1 were considered differentially expressed [61]. Sample and miRNA clustering based on Euclidian distances was performed using the gplots R package, and the clustering results were displayed graphically as MA plots and heatmap dendrograms. For the qRT-PCR and western blot data, Student's t-test was used for independent variance to determine significance (*p* < 0.05). Statistical analysis and plotting of the data was performed using MS Excel software for Windows, and the results were presented as mean relative expression values ± standard error of the mean (SEM).

## SUPPLEMENTARY MATERIAL TABLES AND FIGURE


