# Supplementary material for: Circadian disruption-induced microRNAome deregulation in rat mammary gland tissues
Source: Oncoscience. 2015 Apr 26;2(4):428–42. doi: 10.18632/oncoscience.157 (PMC4468328; doi:10.18632/oncoscience.157)

Circadian disruption-induced microRNAome deregulation in rat mammary gland tissues

Supplementary Table S1: Acute photoperiod-shifting schedule.

DAY	
1	Lights off at 13:30
2	Lights off at 10:30
3	Lights off at 07:30
4	Lights off at 04:30
5	Lights off at 01:30
6	Lights off at 22:30

Supplementary Table S2: Chronic photoperiod-shifting schedule.

DAY		DAY	
1	Lights off at 13:30	33	Lights off at 19:30
2	Lights off at 10:30	34	Lights off at 16:30
3	Lights off at 07:30	35	Lights off at 13:30
4	Lights off at 04:30	36	Lights off at 10:30
5	Lights off at 01:30	37	Lights off at 07:30
6	Lights off at 22:30	38	Lights off at 04:30
7-16	Re-entrainment-lights off at 19:30	39-48	Re-entrainment-lights off at 01:30
17	Lights off at 16:30	49	Lights off at 22:30
18	Lights off at 13:30	50	Lights off at 19:30
19	Lights off at 10:30	51	Lights off at 16:30
20	Lights off at 07:30	52	Lights off at 13:30
21	Lights off at 04:30	53	Lights off at 10:30
22	Lights off at 01:30	54	Lights off at 07:30
23-32	Re-entrainment-lights off at 22:30		

Supplementary Figure S1: Flow chart illustrating the breakdown of the circadian disruption tissue extraction groups.

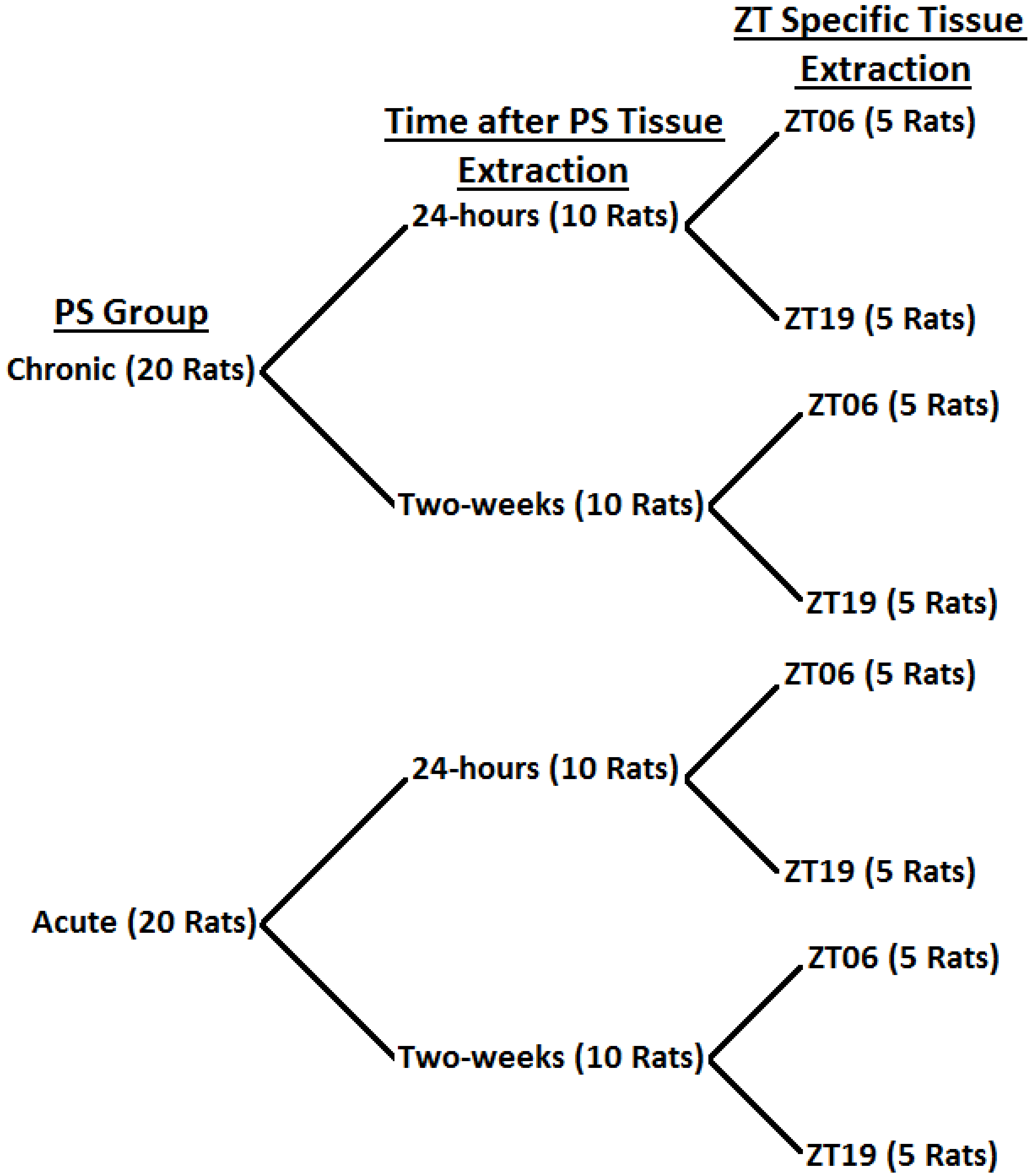

Supplement: Supplementary file 1 [file oncoscience-02-0428-s001.pdf]
